# Supplementary figures and images for: The pretreatment albumin to globulin ratio predicts survival in patients with natural killer/T-cell lymphoma
Source: PeerJ. 2016 Mar 3;4:e1742. doi: 10.7717/peerj.1742 (PMC4782740; doi:10.7717/peerj.1742)

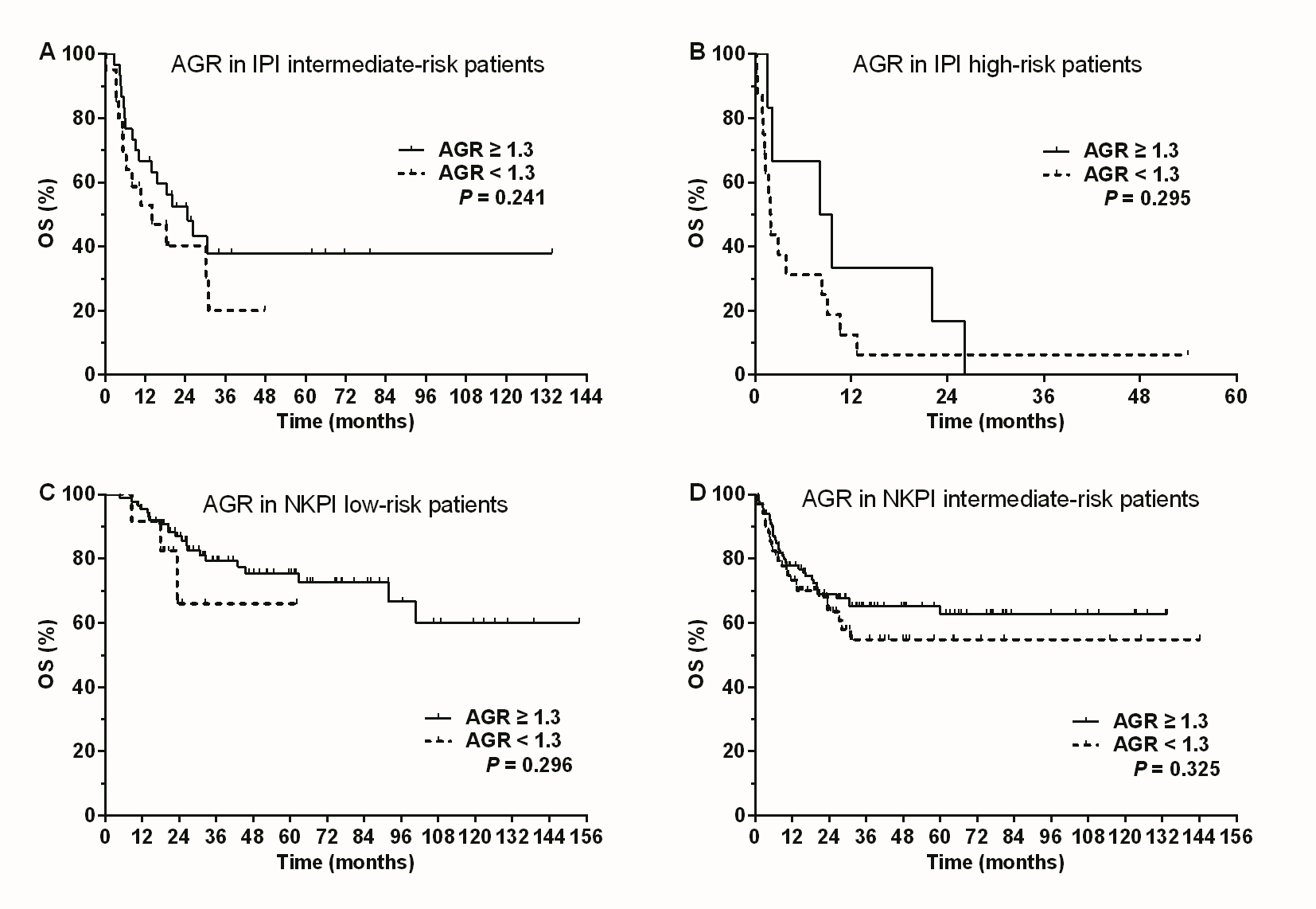

Supplement: Figure S1 — Abbreviations: OS, overall survival. [file peerj-04-1742-s001.png]
